# Adherence to exercise interventions in older people with mild cognitive impairment and dementia: A systematic review and meta-analysis

**DOI:** 10.1016/j.pmedr.2020.101139

**Published:** 2020-06-01

**Authors:** Claudio Di Lorito, Alessandro Bosco, Vicky Booth, Sarah Goldberg, Rowan H. Harwood, Veronika Van der Wardt

**Affiliations:** a Division of Rehabilitation, Ageing and Wellbeing, School of Medicine, University of Nottingham, Queen’s Medical Centre, Nottingham NG7 2UH, United Kingdom; b Division of Psychiatry and Applied Psychology, School of Medicine, University of Nottingham, Institute of Mental Health, Triumph Road, Nottingham NG7 2TU, United Kingdom; c School of Health Sciences, University of Nottingham, Queen’s Medical Centre, Nottingham NG7 2UH, United Kingdom; d Wissenschaftliche Mitarbeiterin, Zentrum für Methodenwissenschaften und Gesundheitsforschung Abteilung für Allgemeinmedizin, Präventive und Rehabilitative Medizin, Philipps-Universität Marburg Karl-von-Frisch-Straße 4, 35032 Marburg, Germany

**Keywords:** Systematic review, Mild cognitive impairment, Dementia, Adherence, Physical exercise, Physical activity

## Abstract

Adherence to physical exercise is associated with multiple benefits in people with mild cognitive impairment (MCI) and dementia. Given the gap in research, this systematic literature review aimed to determine in the context of exercise intervention studies for people with MCI and dementia: 1. How adherence is defined, monitored and recorded; 2. Adherence rates; 3. Attrition, compliance and adverse events and 4. Intervention characteristics associated with adherence. Embase, Medline, PsychInfo, SPORTDiscus, AMED, CINAHL and the International Bibliography of Social Sciences were searched in November 2018. The data were analyzed through descriptive and correlation/inferential statistics. Forty-one studies were included, 34 involving participants with dementia (n = 2149) and seven participants with MCI (n = 970). Half of the studies operationally defined adherence. Mean adherence rate was 70% [CI, 69–73%]. Adherence was significantly associated with endurance/resistance training, and interventions not including walking. The review found a lack of consistency around reporting of adherence and of key variables mediating adherence, including compliance, attrition and adverse events. Further research using more reliable measures is needed to confirm whether a correlation exists between length of interventions and adherence in participants with MCI and dementia and to identify the factors or strategies that mediate adherence in this population. Relevant implications for practice include a consideration in the development of new interventions of elements associated with higher adherence in this review, such as endurance/resistance training, and the provision of exercise in group formats.

## Highlights

• This literature review is on adherence to exercise interventions.
• It focuses on people with dementia and mild cognitive impairment (MCI)
• Forty-one studies are included in the review.
• Mean adherence is 70%. It does not differ between participants with dementia and MCI.
• Interventions with endurance/resistance elements yield higher adherence.

## 1 Introduction

The population is aging rapidly, with estimates reporting that by 2050, nearly 2 billion (22%) individuals worldwide will be 60 years old and over (World Health Organisation, 2017). These numbers represent a public health priority in view of the high prevalence of chronic disease, physical and mental health problems of aging individuals (Blondell et al., 2014). Cognitive decline associated with aging represents a major issue.

The association is not exclusive of normal brain deterioration typically occurring in healthy individuals, but it is also found in clinical conditions, such as Mild Cognitive Impairment (MCI) or dementia (Miller et al., 2012, Eric Ahlskog et al., 2011). Dementia is a syndrome causing deterioration in memory, thinking, behavior and the ability to perform everyday activities (World Health Organisation, 2017). MCI is characterized by deteriorated cognition without a significant impact on daily activities (Alzheimer’s Society, 2019).

MCI and dementia are interlinked, with a rate of transition from mild impairment to dementia of 10–15% annually and of 50% in 5 years (Rosenberg et al., 2006). MCI and dementia also share similar risk factors, some non-modifiable (e.g. age, genetic makeup), and others that can be changed through preventative measures (Molinuevo et al., 2010, Aarsland et al., 2010). Social, physical and mentally stimulating activities targeting various vascular and lifestyle-related risk factors may be protective against dementia (Solomon et al., 2014, Mangialasche et al., 2012). For individuals who have developed the condition, engaging in regular exercise may present multiple benefits on executive functioning, mobility, activities of daily living, independence, and quality of life (Karssemeijer et al., 2017, Öhman et al., 2016, Pitkälä et al., 2013, Blankevoort et al., 2010, Forbes et al., 2015, Potter et al., 2011, Heyn et al., 2004, Hauer et al., 2012, Hoffmann et al., 2016, Lamb et al., 2018, Lowery et al., 2014, Prick et al., 2017, Rolland et al., 2007, Schwenk et al., 2010, Schwenk et al., 2014, Suzuki et al., 2012, Telenius et al., 2015).

To obtain the continued health benefits associated with exercise, adherence is key (Robison and Rogers, 1994). Adherence can be intended as *‘maintaining an exercise regimen for a prolonged period following the initial adoption phase’* (Lox et al., 2016). A six-month home-based exercise intervention for people with dementia found that participants who adhered to ≥70% to the prescribed regime had significantly better balance at follow-up than those who adhered <70% (Taylor et al., 2017).

Given its importance, adherence guidelines have been set around exercise for older adults. The World Health Organisation (WHO) recommend that older adults engage in at least 150 min of moderate-intensity aerobic exercise or 75 + minutes of vigorous-intensity aerobic exercise per week (World Health Organization, 2010). Older adults who cannot exercise due to health conditions, should engage in physical activity which is commensurate to their abilities as much as possible (World Health Organization, 2010). The UK Chief Medical Officers' Physical Activity Guidelines state that even minimal level of exercise (e.g. walking) generates some health benefits, as opposed to being sedentary (Gibson-Moore, 2019). However, research found poor adherence to exercise by older adults (Jancey et al., 2007, Nyman and Victor, 2011, Hawley, 2009).

In addition, adherence alone, does not necessarily produce positive intervention outcomes, which can be affected by a number of factors, including compliance and adverse events. Compliance is defined as *‘conformity to a prescribed or self-prescribed fitness program’* (e.g. whether the participants exercised at the prescribed intensity, such as heart rate) (Exercise compliance, 2012). Non-compliance can cause a lack of improvement in study outcomes, despite good adherence. Adverse events are defined as *‘untoward medical occurrences that may present during treatment (…), but which do not necessarily have a causal relationship with this treatment’* (e.g. physical ailments) (Uppsala Monitoring Center, 2020). Adverse events can cause the participants to withdraw from an intervention program before completion, a phenomenon defined as ‘attrition’ (Murray et al., 2013), and prevent them from obtaining the associated positive benefits, despite good adherence.

Although adherence has been investigated in a few studies focusing on exercise interventions for people with MCI and dementia (Lam et al., 2015, Tak et al., 2012), there is no literature review synthesizing the current evidence, which also identifies crucial factors such as compliance, attrition and adverse events. Considering this gap in research, the aim of this systematic review aims to fill this gap in research by investigating in exercise interventions studies for older people with MCI and dementia:

1) How adherence is defined, monitored and recorded;
2) Adherence rates;
3) Attrition, compliance and adverse events;
4) Intervention characteristics (i.e. type, length, format, intensity, frequency, duration, setting, incentives for participants) associated with adherence.

## 2 Methods

This review complied with the guidelines of the Preferred Reporting Items for Systematic Reviews and Meta-Analyses (PRISMA) Statement (Moher et al., 2009). The review’s protocol was published on the international database of prospectively registered systematic reviews in health and social care (PROSPERO) (Di Lorito et al., 2018). The search strategy (Appendix A) was based on the PICO (Population, Intervention, Comparison, Outcome) worksheet for conducting systematic reviews (Haynes et al., 1997), which identified three search domains: population (i.e. people with MCI or dementia), intervention (i.e. physical activity, exercise, or sport) and outcomes (i.e. adherence). In developing the search strategy, the research team was assisted by a librarian from the University of Nottingham, with expertise in systematic search of the literature. Minor changes to the search strategy were made to adapt it to the different characteristics of the databases.

Seven databases from relevant disciplines (i.e. medicine, sport, psychology, social sciences) were searched: Embase, Medline, PsychInfo, SPORTDiscus, AMED, CINAHL and the International Bibliography of Social Sciences. The searches were carried out in November 2018. The reference lists of the included studies and of the literature reviews retrieved through the database searches were screened to identify further eligible studies.

### 2.1 Study selection

After removing duplicates, title and abstract of all the records identified through the initial searches were independently screened by three authors (CDL, AB, VVDW), who eliminated clearly ineligible studies. Each of the three authors then independently screened the full texts of the remaining studies against the inclusion/exclusion criteria. Any disagreement in the selection process was resolved by consensus.

### 2.2 Inclusion criteria

• Empirical study collecting primary data;
• Study involved people diagnosed with MCI or dementia (any type);
• Study inclusion criteria for age was 65 + years old, or, if lower, the mean age of study participants was at least 70 years old;
• Study tested the effectiveness of an intervention including exercise, defined as *‘planned, structured and repetitive physical activity’* (Caspersen et al., 1985). If the intervention included multiple components (e.g. cognitive stimulation + exercise), adherence rates must have been reported separately for exercise;
• Study reported adherence to the intervention;
• Any type of exercise intervention, any duration, frequency, intensity and mode of delivery (e.g. individual format, group format);
• Any year and language;
• Published or unpublished study (to reduce publication bias).

### 2.3 Exclusion criteria

• Non-empirical study (e.g. literature review), in the presence of which, its reference page is inspected, to identify any primary studies eligible for the review;
• Study on stroke survivors or people with Parkinson’s disease, HIV, Huntington’s disease, multiple sclerosis or subjective memory complaint (i.e. not clinically diagnosed);
• Study on people younger than 65 years old and with a mean age below 70 years old;
• Study on functional ability (activities of daily living) interventions not including an exercise component;
• Study on interventions with multiple components (e.g. exercise + cognitive training) that do not report adherence to the exercise component separately.

### 2.4 Study quality appraisal

Three independent raters (CDL, VVDW and AB) assessed the quality of the included studies. Each article was appraised by one rater only. The Critical Appraisal Skills Program (CASP) checklist for Randomized Controlled Trials (Program, 2019) was adapted, so that the items are relevant to a literature review around adherence. The total possible score of the tool was 13, with higher scores showing higher quality.

### 2.5 Data extraction

Data on study and intervention characteristics, adherence, attrition, compliance and adverse events were extracted into SPSS (Spss, 2016) using a custom designed form. The form was first piloted on a sample of three studies to ensure it captured the relevant information. The data were extracted by the main author (CDL) and checked by a second independent author (AB) to reduce error and bias.

### 2.6 Data analysis

Based on the study objectives, data analysis was carried out on:

1. How adherence was defined, monitored and recorded. This was reported through narrative synthesis and descriptive statistics.
2. Mean adherence weighted by study sample size. A test for heterogeneity was ran to determine whether a *meta*-analysis of the adherence rates from the individual studies was possible. This was carried out through Higgins’ I^2^ Test, which calculates the percentage of variation of adherence rates across studies due to heterogeneity rather than chance (Higgins and Thompson, 2002, Higgins et al., 2003). The thresholds used for the interpretation of I^2^, as per guidelines from the Cochrane handbook for systematic reviews of interventions (Higgins et al., 2019): 0–40% (heterogeneity not important); 30–60% (may represent moderate heterogeneity); 50–90% (may represent substantial heterogeneity); 75–100% (considerable heterogeneity).
  In addition, subgroup analyses were performed on a number of variables that may affect adherence, by selecting and *meta*-analyzing adherence rates from the studies with the relevant variables. The result was then compared with the original mean adherence (i.e. from all the studies), to determine whether the difference was statistically significant (i.e. p < 0.05).
3. Attrition, compliance and adverse events, analyzed through descriptive statistics. Parametric and non-parametric tests (as appropriate) were conducted to test a potential association between these variables and adherence, intervention characteristics (i.e. type, duration, frequency, setting, format of delivery, incentives to adherence), and participants’ characteristics (i.e. cognitive scores, gender and age). P was considered statistically significant if < 0.05.
4. Characteristics (type, duration, frequency, intensity, format, setting, supervision, incentives to adherence) of interventions associated with adherence. These were identified through parametric and non-parametric tests (as appropriate). P was considered statistically significant if < 0.05.

## 3 Results

### 3.1 Study selection

**Fig. 1:**
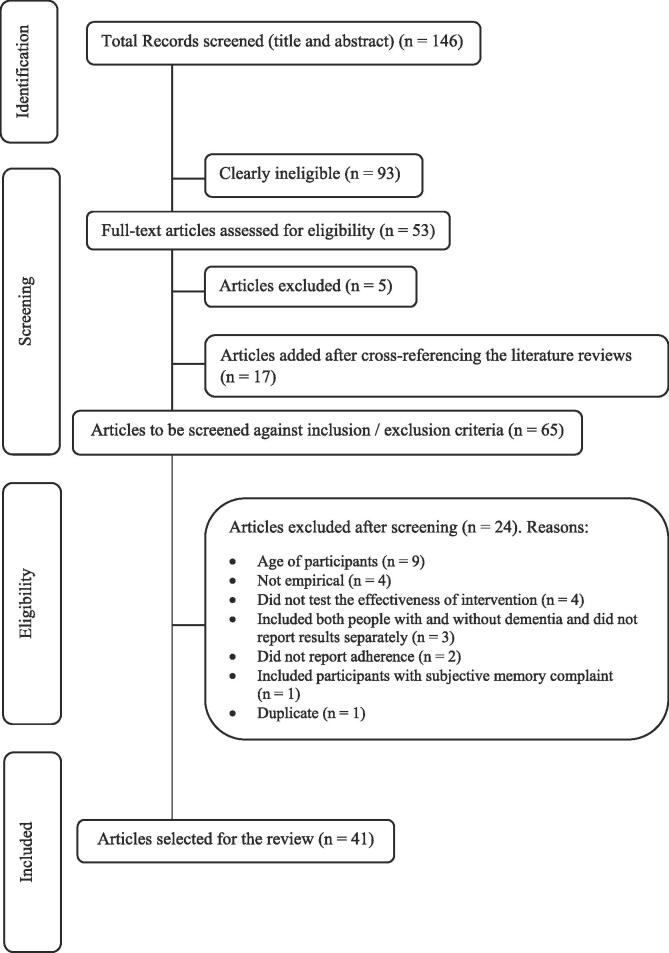
Selection of papers.

The initial search retrieved 146 sources. Of these, 93 were clearly ineligible. Of the remaining 53 studies, five literature reviews were removed and 17 studies added after hand-searching the references of the included literature reviews. The full text of 65 articles was assessed for eligibility against the inclusion/exclusion criteria. Twenty-four of these were excluded and a final number of 41 articles selected for the review. The process is reported in Fig. 1 through a PRISMA flow diagram (Chu, xxxx).

### 3.2 Study quality appraisal

**Fig. 2:**
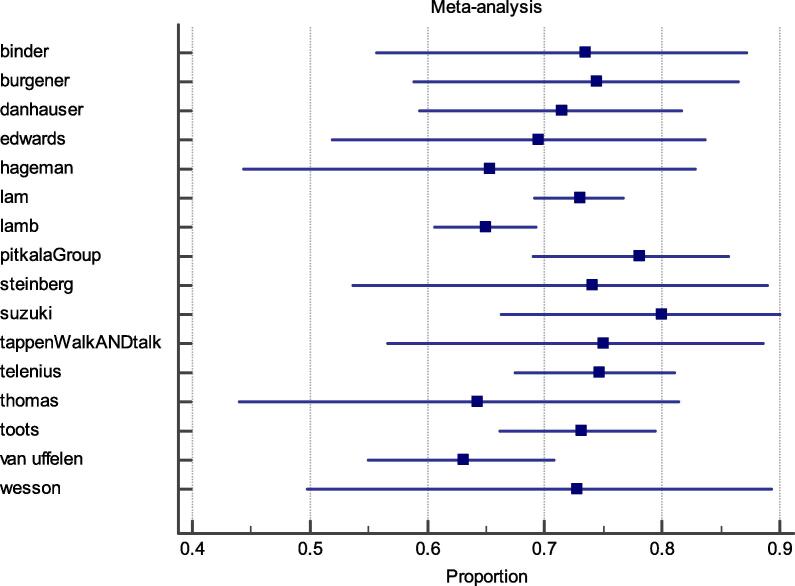
Studies included in *meta*-analysis on adherence rates at the end of the intervention NOTE: The value for Pitkälä^15^ refers to adherence of participants to the group, as opposed to the individual intervention; the value for Tappen^82^ refers to the adherence of participants to the walking plus conversation, as opposed to the walking only intervention.

Results are reported in Table 1. The included studies had a quality score of 5–12 out of 13 (mean = 8; SD = 1). All included studies used an appropriate design and reported the duration of the intervention. Most of the studies did not provide a precise estimate of adherence (n = 10, 24%) or were inconsistent in reporting adherence (n = 11, 27%) (See Fig. 2).

**Table 1:** Study quality appraisal.

Study	Item													
	1	2	3	4	5	6	7	8	9	10	11	12	13	Yes (n)
Arkin (Kuiack et al., 2004)	Y	Y	Y	N	Y	N	Y	Y	Y	N	N	N	N	7
Binder (“Exercise compliance.” Medical Dictionary for the Health Professions and Nursing., 2012)	Y	Y	Y	N	Y	Y	Y	N	Y	N	N	N	Y	7
Bossers (Steinberg et al., 2009)	Y	Y	Y	Y	Y	N	Y	Y	Y	N	N	Y	N	9
Bossers (Folstein et al., 1975)	Y	Y	Y	Y	Y	Y	Y	N	Y	N	N	N	N	8
Brami (Robison and Rogers, 1994)	Y	Y	Y	N	Y	N	N	N	N	Y	N	N	N	5
Brill (Hawley-Hague et al., 2016)	Y	Y	Y	N	Y	N	Y	N	N	N	N	N	N	5
Burgener (Bullard et al., 2019)	Y	Y	Y	N	Y	Y	Y	Y	Y	Y	N	N	Y	10
Cancela (van der Wardt et al., 2017)	Y	Y	Y	Y	Y	N	Y	Y	Y	Y	N	N	N	9
Choi (Peach et al., 2017)	Y	Y	Y	N	N	Y	Y	Y	Y	N	Y	N	N	8
Chu (Apóstolo et al., 2018)	Y	Y	Y	N	Y	N	Y	N	Y	Y	N	N	N	7
Dannhauser (Ybarra et al., 2008)	Y	Y	Y	Y	Y	Y	Y	Y	Y	Y	N	N	Y	11
Edwards (Adolphs, 2009)	Y	Y	Y	Y	Y	Y	Y	Y	Y	Y	N	N	N	10
Hageman (World Health Organization, 2010)	Y	Y	Y	Y	Y	N	Y	N	Y	Y	N	N	N	8
Hauer (Fratiglioni et al., 2004)	Y	Y	Y	N	N	Y	Y	Y	Y	Y	N	N	N	8
Hauer (Fratiglioni et al., 2000)	Y	Y	Y	N	Y	Y	N	N	N	Y	Y	Y	N	8
Hoffman (Jancey et al., 2007)	Y	Y	Y	Y	N	Y	Y	Y	Y	Y	N	N	N	9
Kemoun (Arkin, 2003)	Y	Y	Y	Y	Y	Y	Y	N	N	N	N	Y	N	8
Kuiack (Lox et al., 2016)	Y	Y	Y	N	Y	Y	Y	N	Y	N	N	N	N	7
Lam (Taylor et al., 2017)	Y	Y	Y	N	Y	N	Y	Y	Y	Y	Y	N	Y	10
Lamb (Hageman and Thomas, 2002)	Y	Y	Y	Y	Y	N	Y	Y	Y	N	Y	Y	N	10
Lowery (Teri et al., 1998)	Y	Y	Y	N	Y	Y	Y	Y	N	Y	N	N	N	8
Pitkälä (Blankevoort et al., 2010)	Y	Y	Y	N	Y	Y	Y	Y	Y	N	Y	N	Y	10
Prick (Sobol et al., 2016)	Y	Y	Y	N	Y	Y	Y	Y	Y	Y	Y	N	*	10**
Rolland (Yágüez et al., 2011)	Y	Y	Y	N	Y	Y	Y	Y	Y	Y	Y	Y	N	11
Santana-Sosa (Binder, 1995)	Y	Y	Y	N	Y	Y	Y	N	N	N	N	N	N	6
Schwenk (Lautenschlager et al., 2008, Bossers et al., 2014)	Y	Y	Y	N	N	Y	Y	N	N	Y	N	N	N	6
Sobol (Nyman and Victor, 2011)	Y	Y	Y	Y	Y	Y	Y	Y	N	Y	Y	Y	Y	12
Steinberg (Murray et al., 2013)	Y	Y	N	N	Y	N	Y	Y	N	Y	Y	N	Y	8
Suzuki (Bossers et al., 2015)	Y	Y	Y	Y	Y	Y	Y	Y	N	Y	N	N	Y	10
Tak (Brill et al., 1995)	Y	Y	Y	Y	N	Y	Y	Y	Y	Y	Y	N	Y	11
Tappen (Burgener et al., 2008)	Y	Y	Y	N	Y	Y	Y	N	N	Y	N	N	N	7
Taylor (Cancela et al., 2016)	Y	Y	N	N	Y	Y	Y	Y	Y	Y	Y	Y	N	10
Telenius (Choi and Lee, 2018)	Y	Y	Y	Y	Y	Y	Y	N	Y	Y	Y	Y	Y	12
Teri (Gibson-Moore, 2019)	Y	Y	N	N	Y	Y	Y	N	Y	Y	N	N	N	7
Thomas (Choi and Lee, 2018)	Y	Y	Y	Y	Y	N	Y	N	Y	Y	N	Y	N	9
Toots (Dannhauser et al., 2014)	Y	Y	Y	Y	Y	Y	Y	N	Y	Y	N	N	Y	10
Van Uffelen (Edwards et al., 2008)	Y	Y	Y	Y	Y	N	N	Y	Y	N	Y	Y	N	9
Venturelli (Hauer et al., 2017)	Y	Y	Y	Y	Y	Y	Y	N	N	N	N	N	N	7
Volkers (Hauer et al., 2012)	Y	Y	Y	N	Y	N	N	N	Y	Y	Y	N	N	7
Wesson (Kemoun et al., 2010)	Y	Y	Y	N	Y	Y	Y	Y	N	Y	Y	N	N	9
Yágüez (Hawley, 2009)	Y	Y	Y	N	Y	Y	Y	N	N	N	N	N	N	6
Yes (n)	41	41	38	17	36	28	37	22	28	27	15	10	11**	

1. Did the authors use an appropriate study design to answer their question?

2. Was the duration of the intervention clearly reported?

3. Was the frequency of the intervention clearly reported?

4. Was the intensity of the intervention clearly reported?

5. Was the setting of the intervention clearly reported?

6. Were dropout rates reported?

7. Were diagnoses of dementia/cognitive impairment based on clinical assessments?

8. Were participants representative of the population under investigation (e.g. gender)?

9. Was the number of participants adequate to the study design?

10. Does the study report how adherence was measured?

11. Did the authors account for potential confounding factors in analysis adherence? For example, were sub-analysis by groups or sensitivity analyses performed?

12. How precise was the estimate of adherence? For example, are 95% Confidence Intervals reported?

13. Is the adherence found in the study in line with that reported in other literature” (Is it between 70 and 80%?)

* There are no other comparable data

### 3.3 Study characteristics

Study characteristics are reported in Table 2. The studies were conducted from 1995 to 2018. All were published in peer-reviewed journals, except for two doctoral theses (Chu, xxxx, Volkers, xxxx). Most studies were from the United States of America (n = 11; 27%), the Netherlands (n = 6; 15%) and the United Kingdom (n = 4; 10%). All the studies were in English, except for one (Brami et al., 2018), which was in French.

**Table 2:** Study characteristics (blank boxes indicate that the information was not provided).

Study	Country	Design	N	Eligibility	Male (n); Female (n)	Age mean (SD)	Type of dementia and/or range of cognitive impairment (cognitive score)	Study outcome(s)
Arkin (Kuiack et al., 2004)	USA	Longitudinal	24	Clinical Dementia Rating interview + CERAD (Consortium to Establish a Registry for Alzheimer’s Disease neurological exam protocol)	8;16	78.8	Early to early-moderate stage Alzheimer’s Disease (MMSE 15–29)	Aerobic fitness and duration; upper and lower body strength
Binder[37]	USA	Feasibility	34	Chronic cognitive impairment or a diagnosis of dementia in the medical record; difficulty with transfers or ambulation, or a history of two or more falls in the previous 12 months; ability to ambulate 25 feet or more without assistance	13;21	88.7 (6.9)	Dementia and cognitive impairment (mean MMSE 14.7)	Physical performance
Bossers (Steinberg et al., 2009)	NL	RCT	132	>70; diagnosis of dementia by Dutch dementia diagnosis team; absence of serious health problems; MMSE between 9 and 23; ability to perform the timed up and go test	28; 104	85.7	Alzheimer’s Disease and vascular dementia (MMSE 9–23)	Cognitive and motor function
Bossers (Folstein et al., 1975)	NL	Feasibility	33	≥70 years old; diagnosis of Dementia; not wheelchair bound, able to walk independently ten meters with or without a walking aid	8; 25	85.2	Dementia (mean MMSE 16.5 ± 4.4)	Cognitive and physical function
Brami (Robison and Rogers, 1994)	FR	Feasibility	22	Alzheimer pathology (MMSE≤21); able to walk without technical assistance; absence of visual and/or auditory disorders; no contraindications to the practice of physical activity; oral consent	5;17	84,5 (6.7)	Alzheimer’s Disease	Physical improvements evaluated on the Timed-Up-and-Go (TUG) test and Short Physical Performance Battery (SPPB)
Brill (Hawley-Hague et al., 2016)	USA	Feasibility	10	Ambulatory; diagnosis of dementia; no experience of heart attack or stroke within the last 6 months, unstable angina, or any condition that a physician indicated might be worsened by exercise	2;8	83.0	Dementia (MMSE 5–22)	Strength and flexibility
Burgener (Bullard et al., 2019)	USA	Uncontrolled trial	43	Diagnosis of dementia; score < 2.0 on the Clinical Dementia Rating Scale	23;20	77.9 (7.9)	Early stage dementia (mean MMSE 24.8 ± 3.5)	Cognitive functioning; physical functioning; behavioural outcomes
Cancela (van der Wardt et al., 2017)	ES	RCT	189	> 65 years of age; diagnosis of dementia (DSM IV); able to stand and walk for 30 m without shortness of breath; able to walk safely without assistance; resident of an elderly home-care facility	63;126	82.9 (7.4)	Dementia (mean MMSE 14.9 ± 2.4)	Cognitive decline; memory; depression; functional dependence; neuropsychiatric disturbances
Choi (Peach et al., 2017)	PK	RCT	60	Older adults with mild cognitive impairment; <26 points on the Montreal Cognitive Assessment; ability to communicate; willingness and ability to commit to 6 weeks of intervention	11; 49	74.0	Mild cognitive impairment (<26 on MOCA)	Postural balance; muscle performance; cognitive function
Chu[61]	CA	Feasibility	26	≥65 years old; resident in the nursing home < 6 months; diagnosed with dementia; MMSE score > 10 and < 24; English speaking; able to walk at baseline (with or without gait aids); primary physician deemed participation to be safe; not severely hearing impaired; had a Power of Attorney who consented to participation	5;21	86.8	Severely cognitively impaired and dementia (mean MMSE 15)	Functional mobility; activities of daily living; quality of life
Dannhauser (Ybarra et al., 2008)	UK	Feasibility	70	Diagnosis of Mild cognitive impairment (by two old age psychiatrists and a neuro-Psychologist, based on a full psychiatric assessment, physical examination with an emphasis on neurological examination and a neuropsychological test battery); sedentary lifestyle (no physical exercise two or three times a week for at least 20 min, or active organised sport more than once a week, in the previous six month); at low risk from serious adverse effects from increased physical activity	41;29	74.0	Mild cognitive impairment	Physical health; fitness; cognition
Edwards (Adolphs, 2009)	USA	Feasibility	36	Medical diagnosis of dementia on the medical record; able to follow verbal commands and/or respond to verbal or visual cueing	5;31	85 (5.5)	Moderate to severe dementia (mean MMSE 11.6 ± 5.9)	Negative affect
Hageman (World Health Organization, 2010)	USA	Feasibility	26	Established diagnosis of dementia; attending an adult day care center operated by a local hospital; identified by the nursing supervisor of the center as most likely to benefit from participation; no history of heart attack or stroke within the last six months, or condition that might be worsened by the exercise component	3;23	79.2 (6.6)	Dementia (mean MMSE 18 ± 6.2)	Gait
Hauer (Kuiack et al., 2004)	DE	RCT	122	Having dementia (based on medical history, clinical examination, cerebral imaging, or established neuropsychological test battery); written informed consent; approval by the treating physician and the legal guardian (if appointed); aged 65 and older; ability to walk 10 m without a walking aid; no uncontrolled or terminal neurological, cardiovascular, metabolic, or psychiatric disorder; residence within 15 km of the study center	32;90	82.3 (6.6)	Mild to moderate dementia (mean MMSE 21.7 ± 2.8)	Maximal strength and functional performance
Hauer (Fratiglioni et al., 2000)	DE	RCT	34	MMSE score < 24; age > 65 years; ability to stand or walk 5 m without support; no severe somatic or psychiatric disease; no premature termination of rehabilitation period; residence < 35 kms to the study center; no simultaneous participation in other studies; written informed consent	12;22	81.9 (5.7)	Cognitive impairment (mean MMSE 18.8 ± 4.7)	Functional performance and physical activity
Hoffman (Jancey et al., 2007)	DK	RCT	200	Alzheimer’s Disease (according to the NINDS ADRDA Alzheimer’s Criteria); MMSE score > 19; aged 50–90 years; having a caregiver with regular contact (more than once a month) who was willing to participate in the study; if applicable, on a stable dose of anti-dementia or mood stabilizing medication for 3 months	113;87	70.5	Mild Alzheimer’s Disease (mean MMSE 24)	Cognition (mental speed and attention)
Kemoun (Arkin, 2003)	FR	RCT	31	Diagnosis of Alzheimer by a neurologist based on DSM IV; MMSE < 23; ability to walk 10 m without technical assistance	8;23	81.8 (5.3)	Alzheimer’s Disease (mean MMSE 12.6)	Cognitive function and walking efficiency
Kuiack (Lox et al., 2016)	USA	Case-study	8	Attended a program of daytime respite to caregivers of cognitively impaired adults in University; independently ambulatory; aged 60 years or older; diagnosis of dementia (DSM III R)	3;5	79.0	Dementia (mean MMSE 17)	Muscle strength and power
Lam (Taylor et al., 2017)	HK	RCT	147^1^	MCI; physically stable	113;34	75.4 (6.5)	Mild cognitive impairment (mean MMSE 25.8 ± 2.3)	Cognition (Clinical Dementia Rating sum of boxes (CDR-SOB)
Lamb (Hageman and Thomas, 2002)	UK	RCT	494	Diagnosis of dementia (DSM-IV); MMSE > 10; able to sit on a chair and walk 10 feet (3.05 m) without assistance; living in the community	301;193	77.0 (7.9)	Dementia (mean MMSE 22.1 ± 4.6)	Cognition (Alzheimer’s disease assessment scale-cognitive subscale (ADAS-cog)
Lowery (Teri et al., 1998)	UK	RCT	131	Clinical diagnosis of dementia (ICD-10); at least one significant BPSD symptom defined by the Neuropsychiatric Inventory	57;74	78.0 (7.4)	Dementia (mean MMSE 14.9 ± 8.7)	Behavioural and psychological symptoms of dementia
Pitkälä (Blankevoort et al., 2010)	FI	RCT	210	Aged > 65 years; living with a carer; having a diagnosis of Alzheimer by a geriatrician or neurologist and fulfilling the NINCDS-ADRDA criteria; no diagnosed terminal disease; ability to walk independently with or without a mobility aid; at least 1 fall during the past year, or decreased walking speed, or unintentional weight loss	129;81	77.7 (5.4)	Alzheimer’s Disease	Physical functioning (The Functional Independence Measure (FIM), the Short Physical Performance Battery)
Prick (Sobol et al., 2016)	NL	RCT	111	Diagnosis of dementia made by a physician; 55 years+; living at home with a caregiver willing to participate in the training sessions	70;41	77.0 (7.4)	Dementia (mean MMSE 21 ± 5.19)	Cognitive functioning
Rolland (Yágüez et al., 2011)	FR	RCT	134	Met the National Institute of Neurological and Communicative Diseases and Stroke/Alzheimer Disease and Related Disorders Association criteria for probable or possible AD; lived in the nursing home for at least 2 months; able to transfer from a chair and walk at least 6 m without assistance	34;100	83.0 (7.4)	Mild to severe Alzheimer’s Disease (mean MMSE 8.8)	Activities of daily living (Katz Index of ADLs)
Santana-Sosa (Binder, 1995)	ES	RCT	16	Diagnosed by a trained geriatrician with AD of low-medium grade, i.e., 18 < MMSE < 23; lived in the nursing home for at least 4 months; free of neurological (other than AD), vision, muscle or cardio-respiratory disorders	6;10	76.0 (4.0)	Mild to moderate Alzheimer’s Disease (mean MMSE 20)	Overall functional capacity (muscle strength and flexibility, agility and balance while moving, and endurance fitness)
Schwenk (Lautenschlager et al., 2008)	DE	RCT	61	MMSE score 17–26; >65 years; diagnosis of dementia through (CERAD) test battery; no severe neurologic, cardiovascular, metabolic, or psychiatric disorders; residence within 15 km of the study center; written informed consent (obtained by the patients or by their legal representatives); approval by the treating physician	22;39	81.9 (7.5)	Mild to moderate dementia (mean MMSE 21.4 ± 2.9)	Decrease in performance during dual tasks compared to single task expressed as motor, cognitive, and combined motor/cognitive dual-task cost; gait
Sobol (Nyman and Victor, 2011)	DK	RCT	200	Diagnosis of AD (NINCDS-ADRDA criteria); MMSE ≥20; age between 50 and 90 years; caregiver willing to participate in the study and in contact with participant more than once monthly; if receiving anti-dementia or mood stabilizing medication, dose should be stable for at least 3 months	113;87	70.5 (7.4)	Alzheimer’s Disease (mean MMSE 24 ± 3.6)	Physical performance
Steinberg (Murray et al., 2013)	USA	RCT	27	Probable Alzheimer’s disease based on NINCDS/ADRDA criteria; MMSE > 10; community-residing (not in assisted living); stable medical history and general health; ambulatory; caregiver who spent at least 10 h per week with the participant	8; 19	76.5 (3.9)	Dementia (mean MMSE 20.1 ± 5.1)	Functional performance (e.g. hand function and lower extremity strength)
Suzuki (Bossers et al., 2015)	JP	RCT	50	Living in the community; ≥65 years; having a lower memory in the Logical Memory II subtest of the Wechsler memory scale-revised (WMS-LM II)	27;23	76.0 (7.1)	Mild cognitive impairment (mean MMSE 26.8 ± 1.8)	Cognitive function
Tak[75]	NL	Follow-up to RCT	179	Age between 70 and 80; community dweller; self-reported memory complaints; no report of disability in ADLs; objective memory impairment as measured with a Dutch version of the 10-word learning test; normal cognitive function and absence of dementia as assessed by the Telephone Interview for Cognitive Status; MMSE > 24	101;78	75.1 (2.9)	Mild cognitive impairment (mean MMSE 28.3 ± 1.5)	Recruitment and adherence to programme
Tappen (Burgener et al., 2008)	USA	Uncontrolled trial	71	Clinical diagnosis of probable AD; MMSE < 23; able to stand and walk with the assistance of one individual and/or an assistive device; physician clearance to participate in the exercise	12;59	87.0	Dementia (mean MMSE 10.8)	Functional mobility
Taylor (Cancela et al., 2016)	AU	Uncontrolled trial	42	60 + years; living in the community; clinical diagnosis of dementia (made by a geriatrician or psycho-geriatrician); attending a specialty clinic (e.g. Cognitive Disorders Clinic, Memory Clinic, or Aged Care Clinic) or known to dementia services in the local community; having a carer for a minimum of 3.5 h a week; MMSE > 12/30	20;22	83.0 (7.0)	Mild to moderate dementia (mean ACE-R score 58 ± 14)	Balance (measured by sway on floor and foam) and affect (measured by the 15-item Geriatric Depression Scale (GDS))
Telenius (Choi and Lee, 2018)	NO	RCT	170	> 55 years of age; mild or moderate dementia as measured by the Clinical Dementia Rating scale; able to stand up alone or by the help of one person; able to walk 6 m with or without walking aid	45;125	86.7 (7.4)	Mild to moderate dementia	Balance
Teri[33]	USA	Cross-sectional	30	Meeting the National Institute of Neurologic and Communicative Diseases and Stroke and the Alzheimer's Disease and Related Disorders Association (NINCDS-ADRDA) criteria for probable or possible Alzheimer's; community-dwelling; ambulatory; has an actively involved caregiver living with them	22;8	78.7 (6.4)	Alzheimer’s Disease (mean MMSE 17.8 ± 6.0)	Physical performance
Thomas (Choi and Lee, 2018)	USA	Feasibility	28	>70 years old; diagnosis of dementia in medical record; user in attendance at day care center; no experience of a heart attack or stroke within the last 6 months, or condition that might be worsened by the exercise; able to independently ambulate with or without an assistive device for 10 m	4;24	80.0 (5.6)	Dementia (mean MMSE 17.8 ± 7.2)	Strength and physical function
Toots (Dannhauser et al., 2014)	SE	RCT	186	Aged 65 and older; dementia diagnosis (DSM IV); MMSE 10+; dependent in ADLs; ability to stand up from a chair with armrests with assistance from no more than one person; able to speak Swedish	45;141	85.1 (7.1)	Dementia	Independence in activities of daily living and balance
Van Uffelen[80]	NL	RCT	152	Aged 70–80; memory complaints; objective memory impairment; normal general cognitive function; intact daily functioning; absence of dementia; being able to perform moderate intensity physical activity without making use of walking devices; not using vitamin supplements/vitamin injections/drinks with folic acid, vitamins B-12 and B-6; not suffering from epilepsy, multiple sclerosis, Parkinson’s disease, kidney disorder requiring haemodialysis, psychiatric impairment; not suffering from depression; not using medication for rheumatoid arthritis or psoriasis; no alcohol abuse; not currently living in a nursing home or on a waiting list for a nursing home	85;67	75.0	Mild cognitive impairment (mean MMSE score 29)	Cognitive function measured by neuropsychological tests
Venturelli (Hauer et al., 2017)	IT	RCT	24	≥65 years of age; dependent on assistance in 2 or more personal ADLs; 5 < MMSE < 15; absence of mobility limitations; minimum score of 23 on Performance Oriented Mobility Assessment (POMA) index; constant oxygen saturation during walking (SpO2 > 85%); later stage dementia based on Clinical Dementia Rating Scale		84.0 (5.0)	Late stage dementia	Functional and cognitive decline
Volkers (Hauer et al., 2012)	NL	RCT	148	MMSE < 25; no personality disorders, cerebral traumata, hydrocephalus, neoplasm, disturbances of consciousness and focal brain disorders	36;112	82.0 (7.2)	Mild cognitive impairment and dementia (mean MMSE 15.3 ± 5)	Cognition
Wesson (Kemoun et al., 2010)	AU	Feasibility	22	Community dwelling; >65 years of age; a specialist diagnosis of dementia or ACE-R score ≤ 82; a non-paid carer with a minimum of 3.5 h per Week; English speaking	13;9	78.7 (4.2)	Mild dementia (mean MMSE 24.5 ± 3.1)	Psychological fear of falling
Yágüez (Hawley, 2009)	UK	Feasibility	27	AD diagnosis (ICD-10); 12 < MMSE < 29	11;16	70.5 (8.0)	Alzheimer’s disease (mean MMSE 22.1 ± 3.5)	Cognition

1 Participants receiving the exercise intervention.

More than half of the studies were randomized controlled trials (RCTs) (n = 23; 56%), more than a quarter (n = 11; 27%) were feasibility studies, three studies were uncontrolled trials (7%), one study was longitudinal (2%), one a case-study (2%), one a cross-sectional study (2%) and one a follow-up to an RCT (2%). The sample size greatly varied, based on the study design. It ranged from eight participants from the only case-study included in the review (Kuiack et al., 2004) to 494 participants from a large RCT (Lamb et al., 2018). The mean sample size was n = 92 (SD = 92). The total number of participants with MCI was 970 and 2149 participants were living with dementia.

The eligibility criteria to take part in the studies usually included age, a formal (i.e. clinical) diagnosis of dementia or MCI, and the ability to engage in physical activity. The age of the samples ranged from 70 to 89 (x̅ = 80; SD = 5). The sample under investigation included participants at different stages of any type of dementia (n = 34; 83%) or with MCI only (n = 7; 17%). Mini Mental State Examination (Folstein et al., 1975) cores were not reported in 12 studies (29%). The overall mean MMSE score, weighted by the number of participants per study was 21/30 (SD = 5). The weighted MMSE score mean for participants with MCI only (n = 970) was 27/30 (SD = 2), while for participants with dementia (n = 2149) was 19/30 (SD = 5).

The outcomes of the studies were: physical functioning (n = 28; 68%); cognition (n = 17; 41%); psychological outcomes (n = 5; 12%); behavior (n = 2; 5%); and adherence to the intervention (n = 2; 5%).

### 3.4 Intervention characteristics

The study interventions characteristics are reported in Table 3. The interventions were either purely based on exercise (n = 35; 85%) or delivered in combination with psychoeducation (n = 3; 7%), cognitive activities (n = 2; 5%), social activities (n = 1; 2%) or home hazard reduction (n = 1; 2%). The interventions based on exercise were fitness/ aerobic exercises (n = 17; 41%); exercises for coordination, balance and flexibility (n = 17; 41%); strength exercises (n = 16; 39%); endurance/resistance training (n = 14; 34%), including activities to increase muscular endurance or strength using free weights, bands, body weight or machines; and walking (n = 11; 27%).

**Table 3:** Intervention characteristics, as reported by the authors (Blank boxes indicate that the information was not provided).

Study	Type	Duration (weeks)	Frequency (times per week)	Intensity	Setting	Format of delivery/supervisor	Incentive to adherence
Arkin (Kuiack et al., 2004)	Fitness workout, including stretching and balance exercises, 20 to 30 min of aerobics divided between a treadmill and a stationary bicycle, and 20 to 30 min of upper- and lower-body strength training on five weight resistance machines. Memory- and conversation-stimulation activities during the fitness workout. One session per week of brisk walking incorporated into a community volunteer service or recreational activity	80	2		Community	Individual/Student researchers	Transportation to and from location; participants’ adherence affected researcher’s grade
Binder (“Exercise compliance.” Medical Dictionary for the Health Professions and Nursing., 2012)	50–60 min group activity fitness workout including warm-up and cool-down flexibility exercises integrated into the beginning and end of each session for 5 to 10 min each, straight-leg raises and knee extension exercises, resisted knee extension, ankle extension, and arm exercises using Thera bands	8	3	Below a maximum heart rate of 115 bpm	Nursing home	Group/Therapist	
Bossers (Kuiack et al., 2004)	Thirty-minute strength and walking sessions. Strength exercises included seated knee extension, plantar flexion through toe raises while holding both hands of the trainer, hip abduction by moving the straight leg sideways while standing behind and holding onto a chair, and hip extension by moving the straight leg backward while standing behind and holding onto a chair	9	4	Moderate to high (i.e. rate of perceived exertion (RPE) score 12–15 and 50–85% of maximum heart rate	Nursing home	Individual/Student researchers	
Bossers (Folstein et al., 1975)	Combined aerobic and strength training program. Walking session took part in the corridors of the nursing home or on paved outdoor walking paths near the nursing home. Strength sessions took part in the patients’ rooms and included: (1) seated knee extension, (2) plantar flexion through toe raises, while holding both hands of the trainer, (3) hip abduction by moving the straight leg sideways, while standing behind and holding on to a chair, and (4) hip extension by moving the straight leg backwards, while standing behind and holding on to a chair	6	5	Moderate to high (i.e. rate of perceived exertion (RPE) score 12–15	Nursing home	Individual/Student researchers	
Brami (Robison and Rogers, 1994)	Virtual dance performance (Dance Central on Xbox One). Each session lasted 45 min and was divided into three parts: a warm-up (10 min), the performance of several choreographies (30 min), a return to calm (5 min)	16	1.5	Moderate (i.e. above 40% of reserve heart rate)	Nursing home	Individual	
Brill (Hawley-Hague et al., 2016)	20 min sessions comprising warm-up exercises (Neck stretch, arm reach, should shrugs, shoulder circles, reach to toes), strength (Ball squeeze, chair stand, knee bends), Thera bands (chest press, bicep curls), cool-down (Reach to toes, arm reach, shoulder circles, should shrugs, neck stretch)	11	3		Nursing home	Group/Trainer	Participants were awarded a star which was placed by their name on the attendance Board; participants were given gifts
Burgener (Bullard et al., 2019)	One-hour Taiji exercises consisting of choreography, dynamic Qigong, standing and sitting meditation	40	3		Community	Group/Trainer	Transportation to and from location, follow-up phone calls
Cancela (van der Wardt et al., 2017)	A minimum of 15 min cycling in a recumbent bicycle geared to a very low resistance	60	7	Low	Community	Individual/Therapist	
Choi (Peach et al., 2017)	One-hour sessions consisting of 10 min of warm-up and 10 min of cool-down activities (massage with a sensory ball, gentle stretching, and deep breathing exercises) and 40 min of ground kayak paddling exercise (i.e. sitting on chairs with and without a balance foam, which increases the challenge by providing an unstable surface)	6	2	Tailored to participant’s ability and measured through rating of Perceived Exertion		Group/Trainer	
Chu (Apóstolo et al., 2018)	Individualised walking regime	16	4		Nursing home	Individual	
Dannhauser[62]	30 to 45-min sessions including walking from home, or if unable to walk, exercise through using an upright exercise bike	12	3	Moderate heart rate intensity (i.e. 65–77% of maximum heart rate, estimated to be<60% of VO2 max), determined for each participant from participant’s predicted maximum heart rate (HRmax = 220–age)	Private home	Individual/not supervised	Telephone calls
Edwards (Adolphs, 2009)	30 min chair-based exercises (lateral neck stretch, head rotation, anterior-posterior neck stretch, shoulder shrug, shoulder stretch, wrist reach, ballerina stretch, overhead stretch with weights, arm curl, shoulder press, lateral shoulder press, toe taps, leg thrusts, hamstring stretch), and walking	12	3	Moderate	Nursing home	Group/Trainer	
Hageman (World Health Organization, 2010)	Progressive resistance lower extremity exercise using Thera Band Each session consisting of a brief warm-up, and 12 Thera-band exercises to target the hip flexors, hip extensors, hip abductors, hip adductors, knee flexors, knee extensors, ankle dorsi-flexors and ankle plantar-flexors	6	3	Moderate	Community	Individual/Trainer	
Hauer (Fratiglioni et al., 2004)	Progressive resistance and functional training	12	2	Sub-maximal (i.e. 70–80% of one repetition maximum)		Group	
Hauer (Fratiglioni et al., 2000)	Postural control, strength and functional home training. Postural balance tasks included standing in progressively challenging positions (side by side stance, semi-tandem stance, tandem stance). Strength exercises targeted basic ADL-related key motor functions, including functional strength (such as ankle lifts, chair rises, and stair rises)	6	7	Adjusted to individuals’ performance levels	Private home	Individual/Carer	Weekly phone contact
Hoffman (Lam et al., 2015)	Building up strength and aerobic exercise including 3 × 10 min on an ergometer bicycle, cross trainer, and treadmill with 2–5 min rest in between	16	3	Moderate to high (i.e. 70–80% of maximal heart rate − 220 - the person’s age)		Group/Therapist	
Kemoun (Fratiglioni et al., 2004)	One-hour sessions consisting of 10 min of contact, articular mobilization and warm-up 40 min of active exercise and 10 min of return to calm and relaxation. The active exercise included either walking and the amelioration of walking parameters through motor route exercises (e.g. walking by striding over boards, going up a step, zigzagging), stamina exercises (i.e. ergo cycle with the arms and the legs) or leisurely physical activities (e.g. dance and stepping) that combined stamina, equilibrium and walking	15	3	Light to moderate (i.e. 60–70% of reserve cardiac frequency, measured through cardio frequency meter)	Nursing home		
Kuiack (Lox et al., 2016)	One-hour sessions comprising 10 min of stretching and flexibility exercises, and then three sets of eight repetitions of five resistance exercises (leg extension/curl, shoulder press/lateral pull, hip abductor/adductor, chest/back and abdomen/back)	12	2		Community	Group/Trainer	
Lam (Taylor et al., 2017)	One-hour session of either stretching & toning exercise, mind body exercise (e.g. Tai Chi) or aerobic exercise (e.g. static bicycle riding)	48	3		Community and private home	Group and individual	If a participant failed to turn up at the training center, the staffs would contact the participants and family members
Lamb (Hageman and Thomas, 2002)	Sixty to ninety-minute session comprising aerobic exercise (static cycling with a five minute warm-up period followed by up to 25 min of cycling) and strength training (arm exercises using hand held dumb bells, including at least a biceps curl and, for more able individuals, shoulder forward raise, lateral raise, or press exercises, and leg strength training exercises using a sit-to-stand weighted vest or a waist belt	48	2	Moderate to high, tailored to participants, using a six minute walk test	Community	Group and individual/Therapist	Behavioural strategies and up to three telephone motivational interviews
Lowery (Teri et al., 1998)	Twenty to thirty minute sessions of walking in the home	12	5	Tailored and based on self-rating of perceived exertion	Private home	Individual/Therapist	Telephone contact
Pitkälä (Blankevoort et al., 2010)	One-hour home exercises addressing the patient’s individual needs and problems in daily functioning or mobility, including climbing stairs, balance training, transfer training, walking, dual tasking, and outdoor activities. Or one-hour group endurance (exercise bikes), balance (walk on a line, training with a bouncing ball, climbing a ladder, getting up from the floor), strength training (leg strength and hip abduction machines) and functioning exercises (throwing a ball as accurately as possible, or doing different functions with the left and right hands while counting numbers forward or backward at the same time)	48	2		Private home and community	Individual or Group/Therapist	Transport to and from venue, peer-support, refreshments
Prick (Sobol et al., 2016)	One-hour session, including strength exercises (Dorsiflexion Knee extension Plantar flexion Hip flexors Knee flexion Hip abduction Hip extension), balance exercises (Transfer exercises from a seated to a standing position, Functional base-of-support Duo exercises), flexibility exercises (Chest stretch Neck stretch Shoulder stretch Ankle stretch Quadriceps stretch) and endurance exercise (walking)	12	<1		Private home	Individual/Trainer	Support through psycho-education, communication skills training and pleasant activities training
Rolland (Yágüez et al., 2011)	Walk, strength (squatting at different levels or repeated stand ups from a chair, lateral elevation of the legs in a standing position, and rising on the toes), balance (small step trial exercises using cones and hoops on the ground and one- or two-leg balance exercises on the ground or on foam-rubber ground sheets), and flexibility training	48	2		Nursing home	Group/Therapist	
Santana-Sosa[71]	Seventy-five-minute sessions including 15-min warm-up and 15-min cool down period of walking without reaching breathlessness (on an inside walking trail) and “gentle” stretching exercises for all major muscle groups; joint mobility exercises focused on shoulder, wrist, hip, knee and ankle joints; resistance training engaging chest, biceps, triceps, shoulder, knee extensors, abductor and adductor muscles, and calf muscles; coordination exercises performed with foam balls of gradually decreasing size over the program, e.g., bouncing a ball with both hands, tossing and catching a ball, etc.	12	3		Nursing home	Individual/Researcher	
Schwenk (Lautenschlager et al., 2008, Bossers et al., 2014)	Two-hour dual-task training and progressive resistance-balance and functional balance training (basic activity of daily living-related motor functions including sitting down and standing up from a chair, standing and walking	12	2	Sub-maximal (i.e. 70–80% of one repetition maximum)		Group/Trainer	
Sobol (Nyman and Victor, 2011)	One-hour sessions, including a general warm up and cool down period, strength training of the lower extremity muscles and aerobic exercise on ergometer bicycle, cross trainer, and treadmill	16	3	Moderate to high (i.e. 70%–80% of maximal hazard ratio (HR: 220 minus the person’s age)	Community	Group/Therapist	
Steinberg (Murray et al., 2013)	Three components: (1) Aerobic fitness: brisk walking; (2) Strength training targeted at major muscle groups, using resistive bands and ankle weights; (3) Balance and flexibility training incorporating shifting center of gravity, tandem walks, forward and backward walks, and chair sit to stands	12			Private home	Individual/Carer	Participants accrued points for performing activities. The goal was to accrue a certain amount of points
Suzuki (Bossers et al., 2015)	Ninety-minute sessions including 10-min warm-up period, 20 min of muscle strength exercise, and 60 min of aerobic exercise, postural balance retraining (e.g. circuit training with stair stepping, endurance walking, and walking on balance boards) and dual-task (e.g. invent their own poem while walking)	48	2	Moderate (i.e. 60% of maximum heart rate)	Community	Group/Therapist	Transportation to and from venue
Tak (Brill et al., 1995)	Two types: (1). Aerobic walking consisting of warm-up, moderate-intensity walking exercise, and a cool down; (2). Non aerobic exercise consisting of introduction, light range-of-motion movements and stretching, and a closing	48	2	Low or moderate (i.e. < 3 or > 3 metabolic equivalents [METs])		Group/Trainer	
Tappen (Bossers et al., 2015)	Thirty minutes of self-paced assisted walking interspersed with rest as needed (with vs. without conversation with supporter)	16	3		Nursing home	Individual/ Student researchers	
Taylor (Cancela et al., 2016)	Exercises were predominantly balance focused, but also included strength and/or combined strength-balance exercises, e.g. tandem stance, knee extensions +/− weights, sit-to-stand, step ups on a block, and sidestepping	24			Private home	Individual/Carer	
Telenius (Choi and Lee, 2018)	Fifty-to-sixty minute sessions including 5 min warm-up, at least two strengthening exercises for the muscle of lower limb and two balance exercises	12	2	High (i.e. 12 repetitions maximum)	Nursing home	Group/ Therapist	
Teri (Gibson-Moore, 2019)	Strength training focused on lower-body strengthening including dorsiflexion (“toe lifts”), knee extension and flexion (“knee straightening” and “back knee bends”), plantarflexion (“toe raises”), hip flexors (“marches”), abduction (“side lifts”), and extension (“back leg lifts”). Balance exercises including transfer exercises (chair stand), base of-support exercises (forward lean), and advanced walking skills (backwards walk). Flexibility training focusing on the back, shoulders, hips, hamstrings, gastrocnemius/soleus/achilles, neck, and hand. Endurance including brisk walking	12			Private home	Individual/Carer	
Thomas (Choi and Lee, 2018)	Resistance training sessions using Thera Band, comprising: a brief warmup, and 12 exercises to target the hip flexors, hip extensors, hip abductors, hip adductors, knee flexors, knee extensors, ankle dorsi-flexors, and ankle plantar-flexors	6	3	Moderate	Community	Individual/Trainer	
Too ts(2014)	Functional exercises (exercises performed in functional, weight-bearing positions similar to those used in everyday situations, such as rising from a chair, stepping up, trunk rotation while standing, and walking) aimed to improve lower limb strength, balance, and mobility	16	2.5	High (i.e. 8- to 12-repetition maximum)	Nursing home	Group/Therapist	
Van Uffelen (Edwards et al., 2008)	Outdoors walking sessions including a warm-up, moderate-intensity walking exercises and cool-down	48	2	Moderate (i.e. > three metabolic equivalents)	Community	Group/Trainer	
Venturelli (Hauer et al., 2017)	30-min aerobic walking	24	4	Moderate	Nursing home	Individual/Carer	Participants were given cookies at the end of each session
Volkers (Hauer et al., 2012)	30-min walking sessions	72	5		Nursing homes and community	Individual/Trainer	
Wesson (Kemoun et al., 2010)	One hour sessions including: (1). Strength training including sit to stand, calf raises and step ups onto a block. (2). Static balance tasks including a series of stance positions with diminishing base of support (i.e. standing with feet together, semi tandem, near tandem and tandem) with eyes open or closed. (3). Dynamic balance exercises including stepping over a strip of matting on the floor, foot taps onto a block, lateral side steps, sideways walking and step ups	12	3		Private home	Individual/Therapist	Phone calls
Yágüez (Hawley, 2009)	Non-aerobic movement-based activity (Brain Gym training) including stretching different parts of the body, circular movements of the extremities and isometric tensions of muscles groups. The exercises require fine motor involvement, balance and eye-hand coordination and they are performed sitting or standing	6	1		Community	Group/Trainer	

Intervention duration varied from six (n = 6; 15%) to 80 weeks (n = 1; 2%), with a mean of 23 weeks (SD = 20). One in three interventions lasted three months (n = 13; 32%). In most cases, the participants were invited to exercise twice (n = 12; 32%) or three times a week (n = 14; 34%). The mean frequency of training required to participants across the studies was 3 times weekly. One fourth of the interventions (n = 10; 23%) required participants to exercise for up to 30 min, one third (n = 13; 30%) between 30 and 60 min and one tenth (n = 4; 9%) for more than an hour. The intensity of the interventions was only reported in 24 studies (58%), which used different strategies to measure it, the most commons being heart rate (n = 5; 21%) and One-Repetition Maximum (n = 4; 17%).

Intervention location included nursing homes (n = 15; 36%), the community (excluding participants’ homes) (n = 13; 32%), and the participants’ private homes (n = 10; 24%). The interventions were delivered to the participants individually (n = 21; 51%), in a group (n = 17; 41%) or in both formats (n = 2; 5%). The sessions were delivered/supervised by gym trainers/coaches/instructors (n = 14; 34%), therapists (e.g. physiotherapists, occupational therapists) (n = 11; 27%), carers (n = 5; 12%) and students/research assistants (n = 4; 10%). The participants were unsupervised in one study (2%). Incentives for intervention adherence (e.g., biscuits upon completion of the session) were reported in 14 papers (58%). The most common included regular phone contact (n = 7; 50%) and transportation to and from exercise venue (n = 4; 29%).

### 3.5 How adherence is defined, monitored and recorded

Results for adherence are reported in Table 4. Adherence was operationally defined in half of the studies (n = 20; 49%) as *“The proportion between the number of sessions attended and the number of sessions offered × 100”.* However, not all studies conformed to this. One study (2%) (Steinberg et al., 2009) measured adherence through the percentage of (personal) goals achieved by the individual participants against the goals set at the beginning of the study. The remaining studies (n = 20; 49%) did not define adherence, but just reported adherence rates. All studies reported adherence rates at the end of the intervention period only (i.e. they did not report adherence at different time points during the intervention).

**Table 4:** Information on adherence.

Study	Definition of adherence	Monitoring of adherence	Recording of adherence	Adherence rate at the end of the intervention (%) [95% Confidence Intervals]
Arkin (Kuiack et al., 2004)	Not defined	Not reported	Not reported	87[70–89]
Binder (“Exercise compliance.” Medical Dictionary for the Health Professions and Nursing., 2012)	Not defined	Not reported	Not reported	75[57–85]
Bossers (Steinberg et al., 2009)	Not defined	Not reported	Not reported	89[82–89]
Bossers (Folstein et al., 1975)	Not defined	Not reported	Training calendar	86[69–89]
Brami (Robison and Rogers, 1994)	(N sessions attended/N sessions offered) × 100	Not reported	Not reported	95[67–89]
Brill (Hawley-Hague et al., 2016)	Not defined	Not reported	Not reported	100[72–89]
Burgener (Bullard et al., 2019)	Not defined	Not reported	Not reported	75[60–85]
Cancela (van der Wardt et al., 2017)	Not defined	Therapist	Attendance sheet	88[82–89]
Choi (Peach et al., 2017)	Not defined	Not reported	Not reported	96[89]
Chu (Apóstolo et al., 2018)	(N sessions attended/N sessions offered) × 100	Researcher	Daily log	93[76–89]
Dannhauser (Ybarra et al., 2008)	(N sessions attended/N sessions offered) × 100	Participants	Log	71[60–81]
Edwards (Adolphs, 2009)	(N sessions attended/N sessions offered) × 100	Researcher	Not reported	68[53–82]
Hageman (World Health Organization, 2010)	Not defined	Not reported	Not reported	66[50–83]
Hauer (Fratiglioni et al., 2004)	Not defined	Not reported	Calendar	93[89]
Hauer (Fratiglioni et al., 2000)	(N sessions attended/N sessions offered) × 100	Participant	Not reported	95[81–89]
Hoffman (Jancey et al., 2007)	(N sessions attended/N sessions offered) × 100	Not reported	Training log	84[78–88]
Kemoun (Arkin, 2003)	Not defined	Not reported	Not reported	90[75–89]
Kuiack (Lox et al., 2016)	Not defined	Not reported	Not reported	100[67–89]
Lam (Taylor et al., 2017)	(N sessions attended/N sessions offered) × 100	Members of staff	Not reported	75[71–78]
Lamb (Hageman and Thomas, 2002)	Not defined	Researcher	Attendance log	65[61–69]
Lowery (Teri et al., 1998)	Not defined	Carer	Diary	30[20–42]
Pitkälä (Blankevoort et al., 2010)	(N sessions attended/N sessions offered) × 100	Not reported	Not reported	81[75–86]
Prick (Sobol et al., 2016)	(N sessions attended/N sessions offered) × 100	Participants	Daily log	15[10–23]
Rolland (Yágüez et al., 2011)	(N sessions attended/N sessions offered) × 100	Not reported	Not reported	33[25–41]
Santana-Sosa (Binder, 1995)	Not defined	Not reported	Not reported	98[72–89]
Schwenk (Lautenschlager et al., 2008, Bossers et al., 2014)	(N sessions attended/N sessions offered) × 100	Not reported	Not reported	91[82–89]
Sobol (Nyman and Victor, 2011)	(N sessions attended/N sessions offered) × 100	Not reported	Not reported	83[77–87]
Steinberg (Murray et al., 2013)	(Goals achieved/Goals set) × 100	Carer	Diary	75[55–86]
Suzuki (Bossers et al., 2015)	Not defined	Not reported	Attendance sheet	79[65–87]
Tak (Brill et al., 1995)	(N sessions attended/N sessions offered) × 100	Instructor	Not reported	53[46–60] (Kuiack et al., 2004)
Tappen (Burgener et al., 2008)	(N sessions attended/N sessions offered) × 100	Not reported	Not reported	66[54–76]
Taylor (Cancela et al., 2016)	(N sessions attended/N sessions offered) × 100	Participant	Monthly diary	45[31–60] (Kuiack et al., 2004)
Telenius (Choi and Lee, 2018)	(N sessions attended/N sessions offered) × 100	Not reported	Not reported	75[68–81]
Teri (Gibson-Moore, 2019)	(N sessions attended/N sessions offered) × 100	Carer	Daily exercise log	38[22–54**)**
Thomas (Choi and Lee, 2018)	Not defined	Not reported	Not reported	63[42–76**)**
Toots (Dannhauser et al., 2014)	Not defined	Therapist	Attendance form	73[66–78**)**
Van Uffelen (Edwards et al., 2008)	(N sessions attended/N sessions offered) × 100	Not reported	Not reported	63[54–70**)**
Venturelli (Hauer et al., 2017)	Not defined	Not reported	Not reported	93[74–89**)**
Volkers (Hauer et al., 2012)	Not defined	Not reported	Not reported	21[15–28**)**
Wesson (Kemoun et al., 2010)	(N sessions attended/N sessions offered) × 100	Therapist	Field note	72[52–87**)**
Yágüez (Hawley, 2009)	Not defined	Not reported	Not reported	90[72–89] (Kuiack et al., 2004)

Twenty-six studies (63%) did not report who monitored adherence. In the remaining studies, monitoring was equally performed by the study participants (i.e. self-reporting) (n = 4; 10%), the participants’ carers (e.g. family members or members of staff in nursing homes) (n = 4; 10%), the professionals delivering the intervention (e.g. gym instructors, physiotherapists, occupational therapists) (n = 4; 10%), and the study researchers (n = 3; 7%).

Twenty-six studies (63%) did not report how adherence was recorded. In the remaining cases, attendance sheets/forms, training logs and calendars were more frequently used (n = 11; 27%) than diaries (n = 3; 7%) and field notes (n = 1; 2%).

### 3.6 Adherence rates

Adherence rates for each study are reported in Table 4. Overall, adherence rates ranged from 16% to 100%, with a mean adherence of 70% (SD = 21). The Higgins’ I^2^ Test revealed a high level of heterogeneity (I^2^ = 95%; 95% C.I. 94–96). Results from the subgroup analyses are reported in Table 5. None of the subgroup analyses evidenced any statistically significant difference with the original adherence rate mean (i.e. all studies). The highest adherence was found for studies which required participants to train more than three times a week (75.0%) and the lowest for studies with including participants exercising in nursing homes (65.4%).

**Table 5:** Sub analyses.

Type of studies included in the sensitivity analysis	Adherence mean resulting from sensitivity analysis (%)	Original adherence rate mean (%)
Studies with incentives to adherence	72.1	70.0
Studies where participants were unsupervised	71.0	
Studies where participants did not have to travel to participate in the intervention	73.1	
Studies with interventions lasting >24 weeks	72.2	
Studies which required participants to train more than three times a week	75.0	
Studies including only participants with dementia	70.3	
Studies including participants with cognitive impairment only	70.5	
Studies including participants exercising in nursing homes	65.4	
Studies including participants exercising in the community	70.1	
Studies including participants exercising in private homes	65.9	
Studies including participants aged ≤ 80 years	69.9	
Studies including participants aged >80 years	71.1	
Studies with attrition rate below total attrition mean (<17%)	74.8	
Studies with attrition rate above total attrition mean (≥17%)	74.6	

Statistically significant differences from the original adherence mean are marked with *

Only one study reported adherence six months following the intervention period (Tak et al., 2012). The study found that more than half of participants had discontinued exercise after the end of the trial, and one quarter had continued. Health complaints, lack of time, injuries and lack of motivation were the most reported reasons for not continuing.

### 3.7 Attrition, compliance and adverse events

Attrition rates at the end of the study intervention were reported in 35 studies (85%). It averaged 17% (SD = 13) of the initial number of study participants. It ranged from 0% to 59%. We did not find any statistically significant association between attrition and adherence, intervention characteristics (i.e. type, duration, frequency, setting, format of delivery, incentives to adherence), and participants’ characteristics (i.e. cognitive scores, gender and age) (p > 0.05).

Adverse events and serious adverse events were reported in 25 studies (61%). In those studies where they were reported, the data were extremely diverse, ranging from no adverse events at all to each study participant experiencing an average of 13 adverse events. Compliance was reported in seven studies only (17%). Again, the data were extremely diverse, ranging from 16% to 100%. The sparse data on adverse events and compliance did not allow us to test their association with adherence, intervention and participants’ characteristics. Details on attrition, adverse events and compliance are fully reported in Table 6.

**Table 6:** Attrition, adverse events and compliance (as reported in individual studies).

Study	N Attrition; % on N participants; (reasons)	N Adverse events; % on N participants; (details)	Compliance (%)
Arkin (Kuiack et al., 2004)	3; 12	2; 8; (serious injuries not related to the project)	100
Binder (Hawley-Hague et al., 2016)	9; 26; (Eight participants refused to perform the exercises, and 1 revoked consent)	None; 0	75
Bossers (Steinberg et al., 2009)			
Bossers (Folstein et al., 1975)	3; 9; (Two not willing to perform the pre-tests, 1 due to injury)	19; 58; (1 injury not related to study, 6 sore leg muscles, 12 sense of exertion)	
Brami (Robison and Rogers, 1994)	9; 41; (One change in care, four change in health status, four not willing to perform the pre-tests)		
Brill (Hawley-Hague et al., 2016)			
Burgener (Bullard et al., 2019)	10; 23; (one change of residence, one illness, three not needing the intervention, three disability, one change in residence, one involvement in other programs)		
Cancela (van der Wardt et al., 2017)	59; 31; (28 deaths, 15 transfers, five health issues, four refusals, three non-adherence, one cognitive deterioration, one due to medication, one hospitalization, one loss of interest)	34 unrelated to study; 18; (28 deaths, five health issues, one hospitalization)	
Choi (Peach et al., 2017)	4; 7; (One insufficient attendance, two did not complete the post-test, and one moved)		
Chu (Apóstolo et al., 2018)	1;4; (death)	331 unrelated to study; mean = 13 per person	
Dannhauser (Ybarra et al., 2008)	3;4; (two due to the time commitment, one due to physical ill health)	2; 3; (unrelated to study, of which one stroke, one fracture of ankle)	>50
Edwards (Adolphs, 2009)	2; 6; (one hospitalization, one death)	2; 6; (one hospitalization, one death)	
Hageman (World Health Organization, 2010)			
Hauer (Fratiglioni et al., 2004)	23; 19; (seven death, nine serious medical events, seven interrupted training and rejection of any additional testing)	16; 13; (unrelated to study, of which seven death, nine serious medical events)	
Hauer (Fratiglioni et al., 2000)	6; 18 (three for medical reasons, two for lack of compliance, one death)	1 unrelated to study; 3; (death)	
Hoffman (Jancey et al., 2007)	10; 5; (two dementia progression, five medical illness, two self-withdrawals, one family illness)	71; 35; (seven related to study, including one atrial fibrillation and six musculoskeletal problems	
Kemoun (Arkin, 2003)	7; 23; (three lost motivation, three had a stroke, one had hallucinations)		
Kuiack (Lox et al., 2016)	3; 37; (unspecified)		
Lam (Taylor et al., 2017)	32; 22; (unspecified)	1 unrelated to study; 1; (death)	
Lamb (Hageman and Thomas, 2002)	76; 15; (45 withdraws, 18 deaths, 13 losses to follow up)	29; 6; (eight related to study. Four serious adverse events related to study, including one hospitalization, two injurious falls, and one case of worsening hip pain)	
Lowery (Teri et al., 1998)	15; 11; (nine withdrew, four lost to follow up, two died)	8; 6; (unrelated to study, including six falls and two deaths)	
Pitkälä (Blankevoort et al., 2010)	56; 27; (17 deaths, 18 admissions to nursing homes, 13 self-withdrawals, 8 deterioration of health)	491; average: two per person; (96 hospital admissions, 365 falls, 17 deaths, 13 fractures)	
Prick (Sobol et al., 2016)	46; 41; (16 carer burden, 13 participant burden, 6 deaths, 6 admissions to nursing homes, 4 carer health)	Six; 5; (deaths unrelated to study)	16
Rolland (Yágüez et al., 2011)	24; 18; (15 deaths, 8 changes of institutions, one self-withdrawal)	297; average: two per person; (275 falls, 15 deaths, seven fractures, and five falls, the latter occurred during exercise)	
Santana-Sosa (Binder, 1995)	None; 0	None; 0	
Schwenk (Lautenschlager et al., 2008, Bossers et al., 2014, Bossers et al., 2015, Brill et al., 1995, Burgener et al., 2008, Cancela et al., 2016, Choi and Lee, 2018)	12; 20; (seven lack of motivation, three deaths, two serious adverse event)	5; 8; (unrelated to study, of which three deaths)	
Sobol (Nyman and Victor, 2011)	11; 5; (four medical illness, four self-withdrawal, two dementia progression, one family illness)	1; 1; (serious adverse event - atrial fibrillation - possibly related to the study). Unspecified number of musculoskeletal problems and dizziness /faintness, half related to the study	80
Steinberg (Murray et al., 2013)		7; 26 (one death, one Syncopal episode, one fractured metatarsal, one transient ischemic attack, one wrist pain, one ganglion cyst, one light-headed post-phlebotomy)	
Suzuki (Bossers et al., 2015)	3; 6; (one medical illness, one refusal and one did not give reasons)		
Tak (Brill et al., 1995)	13; 7; (five problems with walking or moving, four illness or injury, two complaints related to program, one too busy, one intensity too high)		
Tappen (Burgener et al., 2008)	6; 8; (unspecified)		
Taylor (Cancela et al., 2016)	9; 21; (one died, two were placed in residential care, four refused, one was unwell, and one withdrew from the study)		
Telenius (Choi and Lee, 2018)	16; 9; (seven withdrawals, three deaths, four transfers and four illnesses)	None related to study	70
Teri (Gibson-Moore, 2019)	2; 7		
Thomas (Choi and Lee, 2018)			
Toots (Dannhauser et al., 2014)	29; 16; (25 deaths, two transfers, one medical withdrawal, one hospitalization)	1; 1; (death possibly related to study)	75
Van Uffelen (Edwards et al., 2008)	90; 59; (51 illnesses, 15 too busy, six locations too far, six too intensive, one too light, 11 unspecified)	None related to study	
Venturelli (Hauer et al., 2017)	3; 12; (two strokes and one heart failure)	None; 0	
Volkers (Hauer et al., 2012)			27
Wesson (Kemoun et al., 2010)	1; 4; (hospitalization)	4; 18; (stiffness, dizziness and mild joint pain)	
Yágüez (Hawley, 2009)	3; 11		

### 3.8 Characteristics of interventions associated with higher adherence

Non-parametric tests were conducted due to the non-normally distributed data associated with adherence rates. The only meaningful results were:

• Adherence rates were found to be significantly associated with endurance/resistance training (*U* = 132, *p* = 0.05) and with interventions that did not include walking (*U* = 97, *p* = 0.01).
• A negative correlation, though not statistically significant, was found between adherence and intervention duration (Spearman’s rank *r_s_* = −0.24, *p* = 0.11) and between adherence and frequency of training and adherence (*r_s_* = −0.10, *p* = 0.50). This suggests that when the intervention was shorter in duration or less frequent adherence was higher.
• No statistically significant effect was found regarding the format of delivery on adherence (Kruskal Wallis χ^2^(2) = 1.73, p = 0.42), although adherence was higher when the interventions were delivered in group (78%; SD = 17) compared with individual (70%; SD = 25) format.
• No statistically significant effect was found regarding the use of incentives for adherence (*U* = 91, *p* = 0.48), although adherence was higher when the interventions used incentives (82%; SD = 14) compared to when they did not (72%; SD = 22).

## 4 Discussion

This systematic review and *meta*-analysis investigated adherence to exercise intervention studies for older people with MCI and dementia using systematic means of investigations. It found that adherence was calculated similarly across the studies as *‘the proportion between the number of sessions attended and the number of sessions offered, reported in percentage’*. However, less than half of the studies provided a clear operational definition of adherence, which may be due to the fact that adherence was not the primary outcome in 98% (n = 40) of the included studies and as a result it was not discussed in depth. A lack of consensus around the concept of adherence has been reported in previous research (Hawley-Hague et al., 2016). Even more sparsely reported was how adherence was monitored. It is worth noting that among the few studies which discussed adherence monitoring, self-reports from study participants were quite frequent, bearing a potential risk for biased/inaccurate information (e.g. due to social desirable responses). This risk is particularly tangible in the context of people with dementia experiencing memory loss, thus urging adoption of more reliable measures in future research.

The weighted mean adherence for all the included studies was 70%. This is in line with the rate found for older people with chronic conditions and healthy older adults. Bullard et al., for example, found ∼77% adherence among adults with cancer, CVD, and diabetes (Bullard et al., 2019), while Nyman and Victor (Nyman and Victor, 2011) reported an adherence of ≥70% for walking and class-based exercise and 52% for individually targeted exercise in healthy older adults.

The subgroup analyses did not find any statistically significant differences with the original mean adherence. Interestingly, the same adherence was found for participants with dementia and MCI, potentially showing how progression of cognitive deterioration may not be accompanied by reduced adherence to exercise. It might be argued that, in order to exercise, participants with dementia need more supervision from others (e.g. carers, trainers), who may boost their motivation to adhere. This may also potentially explain why older participants (i.e. >80 years), who may require greater support to exercise, had higher adherence rate than younger participants (i.e. ≤80 years).

Findings around duration, frequency and intensity, though not statistically significant, suggest that shorter (in weeks) and less frequent (in weekly sessions) interventions might be easier to adhere to for people with dementia and MCI. We speculate that there might be issues in long-term interventions in the context of dementia, as the condition might entail dramatic changes/shifts in the person’s wellbeing over a short period of time, thus resulting in barriers to adherence. This hypothesis warrants further exploring. Particularly relevant, in the context of MCI and dementia, might be issues such as compliance, adverse events and attrition, which, over time, might thwart willingness and ability to adhere to the prescribed exercise regime. Unfortunately, given the lack of systematic reporting of data around these crucial variables in the included studies, we could not explore further their mediation in adherence rates

The factors or strategies used to promote adherence to exercise interventions were also sparsely reported. The review found that when used, these strategies were linked to higher adherence. We add that these might also be instrumental to motivate participants to remain active, to promote enduring lifestyle change and produce sustained health benefits, once the active intervention is over. Other than the incentives identified in this review, a number of other strategies have been identified in the literature (van der Wardt et al., 2017) include using established behavior change techniques (e.g. motivational interviewing), offering individual supervision/tailoring of interventions to meet participants’ needs and preferences (e.g. enjoyable activities), setting SMART (i.e. specific, measurable, attainable, relevant, time-bound) goals, providing booklets/guidance on exercises, giving phone calls or reminders to participants, addressing exercise barriers, sending out information/newsletters, offering continuous support to clinicians, and delivering the intervention in group settings using music. There is also accumulating evidence on the centrality of the role of carers in ensuring adherence for participants with MCI and dementia, particularly as the conditions progress (Peach et al., 2017). The effectiveness of these strategies, however, remains to be established.

This review found that the more challenging the intervention (i.e. including endurance/resistance training and not including walking), the higher the adherence. This might be explained by the fact that more able people sign up to challenging interventions and/or that these are delivered in more cognitively intact populations with dementia. It might also indicate that physical activities that are less demanding and more likely to be already part of the daily routines of participants (i.e. walking) might make participants less motivated to fully engage.

In line with a recent research on the effectiveness of interventions to prevent frailty in older adults (Apóstolo et al., 2018), this systematic review found that, though not statistically significant, adherence was higher when the intervention was delivered in a group format and in the community (as opposed to the participants’ private home), suggesting that aspects including opportunities for socialisation, competitive behavior, social pressure (e.g. feeling under the scrutiny of others) and/or modelling might promote adherence. Research has found that a group format might also have other benefits on memory, attention and executive processing (Ybarra et al., 2008, Adolphs, 2009, Fratiglioni et al., 2004, Fratiglioni et al., 2000). However, there are potential barriers associated with community-based exercise delivered in group formats. For example, the review found higher adherence rates when participants did not have to travel to exercise venues in the community to participate in the intervention. This suggests that there might be factors impinging on the willingness and ability of people with these conditions to take part in group-delivered exercise programs in the community.

This review was characterized by certain strengths and limitations. To our knowledge, it is the first work summarising the existing evidence around adherence rates in exercise interventions with people with dementia and MCI. This investigation is timely and relevant, since any intervention program aimed at these populations cannot be successful, unless acceptable adherence from participants is achieved. This work was undertaken following standardised operating procedures and reporting systems (PRISMA), which ensure internal validity to study findings. It followed a protocol published in the International prospective register of systematic reviews (PROSPERO) (Di Lorito et al., 2018).

The main limitation of this work was that in all the studies but one (Tak et al., 2012), adherence was not the primary outcome. As a result, adherence, and other important mediating factors including compliance, adverse events, attrition and incentives to participation, were poorly and disparately reported. This prevented us for exploring further some counterintuitive yet interesting findings. For example, adherence was found to be highest when the intervention was delivered by non-professionals (i.e. students/research assistants) and lowest when it was delivered by trained gym instructors. The use of incentives for students supervisors (who would be given full marks only upon completion of the program) (Arkin, 2003) to encourage adherence from participants might have been instrumental in ensuring higher adherence from participants. It could be also argued that the less severe the participant’s presentation of dementia symptoms, the less intensive “training” a supervisor needed, and therefore the students in Arkin’s study (Arkin, 2003) were supervising a population more likely to adhere than the professional supervisors in the other studies. However, it was impossible to bring statistical evidence in support of this hypothesis.

Given the study limitations, further research should:

1. Make clearer use of terminology (i.e. provide the operational definition of adherence used in the study);
2. Investigate adherence rates by means of more reliable measures (i.e. as opposed to self-reports from participants);
3. Establish whether a correlation exists between length of interventions and adherence in participants with MCI and dementia and develop longitudinal studies investigating post-intervention adherence to exercise;
4. Identify and report the factors (i.e. compliance, adverse events, attrition) or strategies (e.g. incentives, group delivery) having an impact on adherence and the processed through which they mediate between adherence and intervention outcomes.

The study also presents important implications for practice. Those who develop and implement exercise interventions for people with dementia and MCI can:

1. Adopt the common operational definition for adherence found across the studies in this review, so that their results are comparable with the existing evidence-base;
2. Use the weighted mean adherence rate found in this review as a threshold for acceptable adherence against which to compare their own intervention rate;
3. Consider in the development of new interventions elements that were associated with higher adherence in this review, such as inclusion of endurance/resistance training, and the provision of exercise in group formats.
4. Devise strategies to mitigate factors that can mediate intervention adherence (e.g. poor compliance, high attrition rates).

## 5 Conclusion

This review highlighted inconsistencies in the existing empirical research on exercise interventions for people with MCI and dementia regarding how adherence is operationally defined, measured and reported. Because adherence (or lack thereof) is so crucial to obtain study outcomes, effective strategies and adequate resources should be deployed to address this issue.

## Funding

This work was funded by the National Institute for Health Research (10.13039/501100000272NIHR) under its Program Grants for Applied Research Program (Reference Number RP-PG-0614-20007).

## 7 Disclaimer

The views expressed are those of the authors and not necessarily those of the National Health Service, the NIHR or the Department of Health and Social Care.

## Appendix A Search strategy

1. Exp Dementia/
2. Exp Alzheimer's Disease/
3. Exp Cognitive Impairment
4. (dement* or alzheimer* or “cognitive impairment”).ti,ab.
5. 1 OR 2 OR 3 OR 4
6. Exp Exercise/
7. Exp Physical Activity/
8. Exp Sport
9. (exercis* or “physical activit*” or sport*).ti,ab.
10. 6 OR 7 OR 8 OR 9
11. Exp Intervention/
12. Exp treatment/
13. Exp therapy/
14. Exp program/
15. 11 OR 12 OR 13 OR 14
16. adherence.ti,ab.
17. 5 AND 10 AND 15 AND 16
